# Efficacy and Safety of Cryoablation in Barrett’s Esophagus and Comparison with Radiofrequency Ablation: A Meta-Analysis

**DOI:** 10.3390/cancers16172937

**Published:** 2024-08-23

**Authors:** Apostolis Papaefthymiou, Benjamin Norton, Andrea Telese, Daryl Ramai, Alberto Murino, Paraskevas Gkolfakis, John Vargo, Rehan J. Haidry

**Affiliations:** 1 Digestive Diseases and Surgery Institute, Cleveland Clinic, London SW1X 7HY, UK; papaefa@ccf.org (A.P.); benjamin.norton@nhs.net (B.N.); telesea@ccf.org (A.T.); a.murino@nhs.net (A.M.); 2 Gastroenterology and Hepatology, University of Utah Health, Salt Lake City, UT 84132, USA; daryl.ramai@hsc.utah.edu; 3 Department of Gastroenterology, “Konstantopoulio-Patision” General Hospital of Nea Ionia, 142 33 Athens, Greece; pgkolfakis@med.uoa.gr; 4 Department of Gastroenterology, Hepatopancreatology and Digestive Oncology, Erasme University Hospital, Université Libre De Bruxelles (ULB), 1070 Brussels, Belgium; 5 Department of Gastroenterology, Cleveland Clinic, Cleveland, OH 44195, USA; vargoj@ccf.org

**Keywords:** Barrett’s esophagus, cryotherapy, cryoablation, radiofrequency ablation

## Abstract

**Simple Summary:**

Cryoablation therapy is an emerging modality in the treatment of Barrett’s esophagus. Our systematic review collected data on this technique to provide evidence of its efficacy and safety and to compare it with the established RFA. The results from twenty-three studies revealed that the complete eradication of dysplasia and intestinal metaplasia was comparable between RFA and cryoablation. More specifically, cryoablation achieved a complete eradication of dysplasia and intestinal metaplasia at rates of 84.2% (95%CI: 79.1–89.3) and 64.1% (95%CI: 49.2–79.0), respectively, whereas 8.3% (95%CI: 4.7–11.9) of cases presented with recurrence. Studies on cryoballoons seem to be more homogenous in terms of dysplasia treatment, complications, and, especially, strictures.

**Abstract:**

Background: The mainstay approach in endoscopic eradication therapy (EET) for dysplastic Barrett’s esophagus (BE) includes the endoscopic resection of visible lesions, accompanied by ablation of the residual metaplastic epithelium. Cryoablation therapy is one such emerging ablation technique in this field. This systematic review with a meta-analysis aims to accumulate pooled data on cryoablation performance in the treatment of patients with BE and to compare this technique to the standard of care radiofrequency ablation (RFA). Methods: The MEDLINE, Cochrane, and Scopus databases were searched until June 2024 for studies evaluating BE management using cryoablation for cumulative results. The primary outcome was the complete eradication of dysplasia (CED) and intestinal metaplasia (CEIM) in BE compared to RFA, while secondary outcomes included the respective pooled rates using cryoablation, recurrence, and adverse events, with a separate analysis for strictures. The meta-analyses were based on a random-effects model, and the results were reported as odds ratios (ORs) with 95% confidence intervals (CIs). Subgroup analyses by type of cryoablation were also performed. Results: Twenty-three studies (1604 patients) were finally included, four of which were comparative. CED and CEIM did not differ significantly between cryoablation and RFA [OR= 0.95 (95%CI: 0.50–1.81) and OR = 0.57 (95%CI: 0.20–1.63), respectively)]. The pooled rates of CED, CEIM, and recurrence after cryoablation were 84.2% (95%CI: 79.1–89.3), 64.1% (95%CI: 49.2–79.0), and 8.3% (95%CI: 4.7–11.9), accompanied by high rates of heterogeneity. Adverse events were noted in 14.5% (95%CI: 9.9–19.2) of cases, and 6.5% (95%CI: 4.1–9.0) developed strictures. In the subgroup analysis, the cryoballoon achieved a reduction in heterogeneity in CED, adverse events, and stricture formation, whereas spray catheters provided homogenous results in terms of recurrence. Conclusions: Cryoablation provides equal outcomes compared to RFA in the treatment of patients with BE, with the cryoballoon achieving relatively homogenous rates of CED and adverse events.

## 1. Introduction

Barrett’s esophagus (BE) represents the primary precursor lesion to esophageal adenocarcinoma (OAC), following a well-documented progression from intestinal metaplasia to low-grade dysplasia, high-grade dysplasia, and intramucosal carcinoma in a proportion of patients [1]. In addition to the overall increase in incidence of OAC, especially in the Western population, the prognosis for patients with esophageal cancer remains poor, with a five-year survival rate below 20%, largely due to late-stage diagnosis [2,3]. These epidemiological insights underscore the need for screening and prevention strategies, particularly in high-risk populations, as we now have a decade of robust evidence to show that BE-related early neoplasia can be successfully treated with minimally invasive EET and ablation techniques [4].

Ablative therapies for BE have become a cornerstone in the management of dysplastic BE following the endoscopic resection of visible lesions. Current international guidelines recommend these techniques to treat non-visible dysplastic mucosa [5,6,7]. Radiofrequency ablation (RFA), hybrid argon plasma coagulation (hAPC), and cryoablation are the ablative modalities currently in routine clinical practice, with RFA being the most widely studied and used modality. However, even after achieving initial successful eradication, these patients remain at risk of recurrence at a rate of 5.2 per 100 PY, especially when there is a previous history of high-grade dysplasia (HGD), and therefore, these patients still need continuous follow-up examinations, as RFA could fail in 10–20% of cases [8]. Additionally, there is not an inconsiderable rate of adverse events, with post-RFA pain reported in up to 95% of procedures, major pain in 64% of procedures, and dysphagia in 83% of procedures [9]. In a recent network meta-analysis of randomized studies, PDT was associated with a theoretical metaplasia regression; however, this intervention is not used anymore due to the high rates of stricture formation [10,11]. Unfortunately, cryoablation was not included in this study due to the absence of randomized controlled studies (RCTs), although there is evidence of lower stricture incidence.

**Figure 1:**
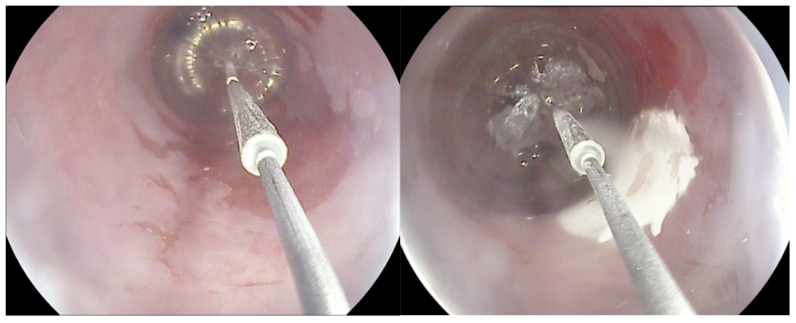
The cryoballoon system.

Cryotherapy for BE encompasses two techniques: cryospray, which uses a through-the-scope spray catheter (truFreeze Spray Cryotherapy System, Steris, Mentor, OH, USA; Generation 2 device, CSA Medical, Baltimore, MD, USA) to deliver Nitrous Oxide (N_2_O), and a cryoballoon (C2 Cryoballoon Focal Ablation system, Pentax Medical, Redwood City, Calif, USA), which can self-adjust its size according to esophagus diameter and delivers Liquid Nitrogen (LN_2_). This minimally invasive procedure involves the application of extremely cold temperatures to destroy abnormal, dysplastic tissue in the esophagus, inducing cellular injury and subsequent cell death (Figure 1). There are limited comparative data between cryoablation and other alternatives; however, the rates of complete eradication of dysplasia (CED) and complete eradication of intestinal metaplasia (CEIM) are similar according to the available studies, and it is hypothesized that the rate of stricture formation might be lower [6,12]. Data are yet to be accumulated and compared in order to provide a clear picture of pooled performance, and safety, on its own and compared to the gold-standard RFA.

This systematic review with a meta-analysis aimed to evaluate the potential differences between cryoablation and RFA, summarize the pooled evidence on cryoablation, and investigate potential variances depending on the specific device.

## 2. Materials and Methods

This study was designed based on the Preferred Reporting Items for Systematic Reviews and Meta-Analysis (PRISMA) guidelines (Supplementary Table S1) [13], and a predefined protocol was registered in the international prospective platform for systematic reviews (PROSPERO Registration Number: CRD42024565043).

### 2.1. Inclusion and Exclusion Criteria

The study’s research question was based on the PICO (population, intervention, control, and outcomes) guidelines and included the evaluation of cryoablation on BE treatment [14]. Considering the comparison between cryoablation and RFA, RCTs and comparative studies, assessing the CED and CEIM, were included when the following prerequisites were met: (A) patients: adult patients (≥18 years old) with BE and dysplasia, with an indication to undergo endoscopic treatment, using (B) interventions: cryoablation (spray catheter or balloon); (C) comparators: patients who received treatment with RFA; (D) outcomes: the complete eradication of dysplasia and intestinal metaplasia after the completion of therapy. Pooled rates of CED, CEIM, BE recurrence, adverse events (AEs), and strictures were also calculated based on any study presenting relative outcomes. Studies with missing data for analysis, without a clear presentation of the results, and not written in the English language, along with case reports or series, and single-arm cohorts, as well as animal studies, were excluded.

### 2.2. Search Strategy

A literature search was performed using the MEDLINE/PubMed, Cochrane, and Scopus databases until 15 June 2024. The search algorithm was modified to fit with each database and included the following search terms: “*Barrett oesophagus*”, “*Barrett dysplasia*”, “*cryoablation*”, “*cryotherapy*”, and “*radiofrequency ablation*”. Other relevant articles were detected by hand-searching the reference lists of the retrieved articles and by using the “similar article” function in PubMed. Anecdotal research and congress proceedings were excluded. In studies with missing data, for our meta-analysis, the authors were contacted. Two independent investigators (AP and BN) selected the respective articles according to the pre-defined inclusion and exclusion criteria, and any disagreements were resolved after consensus with the senior author (RH).

### 2.3. Data Extraction and Quality Assessment

Two investigators (AP and BN) entered data on study characteristics, demographics, intervention details, and stent-related parameters into a form. A third author (AT) reviewed the datasets to identify any discrepancies. Any disagreements were resolved through consultation with a senior investigator (RH).

Quality assessment was independently conducted by two authors (BN and AT) using the ROBINS-I tool, which is applicable to both randomized and non-randomized studies. The quality of non-comparative studies on cryoablation was assessed using the National Heart, Lung, and Blood Institute tool for case series that allows for the evaluation of cohort studies without a comparator [9].

### 2.4. Outcomes

The primary outcome was the rate of CED after the completion of therapy with cryoablation or RFA, documented by follow-up endoscopy with biopsies according to the Seattle protocol or targeted biopsies of visible lesions. Complete eradication of intestinal metaplasia was also investigated between these two groups and assessed with the same method.

The pooled rates of CED and CEIM for cryoablation, assessing the entire literature, were considered as secondary outcomes. The recurrence rates of BE after confirmation of CEIM were also estimated when the respective data from the included studies were provided. Moreover, AEs and specifically stricture formation rates were calculated as well.

Finally, subgroup analyses were performed with regard to the different cryoablation types (spray catheter vs. balloon).

### 2.5. Statistical Analysis

The pooled odds ratios (ORs) and 95% confidence intervals (CIs) for the outcomes were calculated using the DerSimonian and Laird random-effects model, which accounts for both within- and between-study subgroup analyses based on the type of cryoablation, and a sensitivity analysis that included only prospective studies was conducted to investigate and explain the variability in the results. Due to the limited number of studies included in the meta-analysis, a meta-regression analysis was not performed. Publication bias was evaluated through the visual inspection of the funnel plot [15]. A *p*-value of <0.05 was considered statistically significant for all analyses. All statistical analyses were carried out using RevMAN Software (Review Manager, Version 5.4, The Nordic Cochrane Centre, The Cochrane Collaboration, Copenhagen, Denmark) [16].

## 3. Results

### 3.1. Characteristics of Included Studies

**Figure 2:**
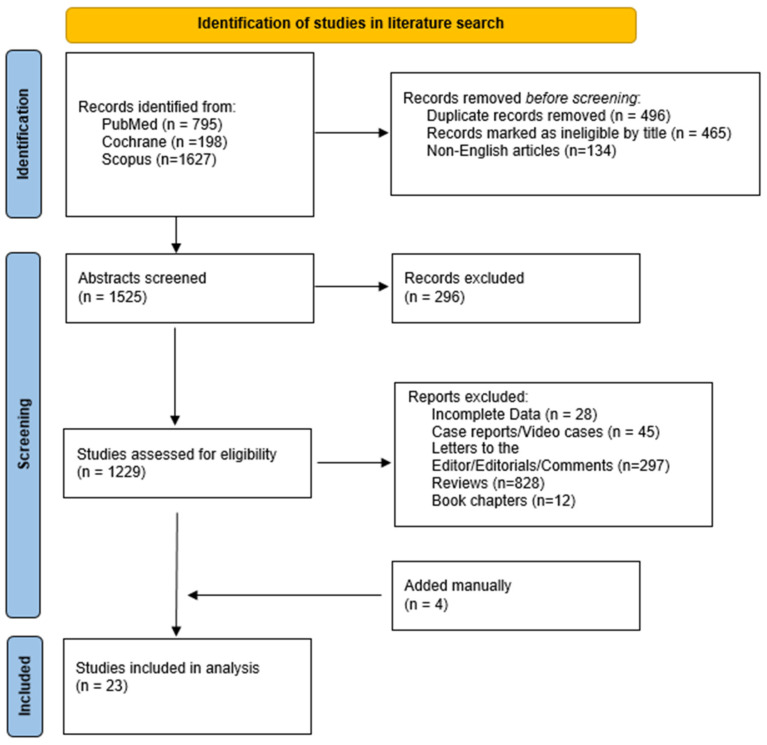
Study flowchart.

The literature search yielded 2620 results. After applying the inclusion criteria, 23 studies [14,15,16,17,18,19,20,21,22,23,24,25,26,27,28,29,30,31,32,33,34,35,36] (1604 patients) were eligible (Figure 2). Table 1 summarizes the main characteristics of the included studies. Nine studies were retrospective [14,23,26,27,29,30,33,34,36], and the remaining fourteen were non-randomized prospective studies [15,16,17,18,19,20,21,22,24,25,28,31,32,35], four of which were multicenter [15,22,24,32]. Twelve studies used a catheter cryoablation device [17,24,25,26,27,28,29,30,31,32,33,35], nine used a cryoballoon system [14,15,16,18,19,20,21,22,23], and two used both of them [34,36]; four studies compared cryoablation with RFA [14,25,26,27]. The vast majority of the studies took place in the USA [14,15,17,19,24,25,26,27,28,29,30,31,32,33,34,35,36], whereas five took place in the Netherlands [16,18,20,21,22] and one took place in different European centers [23].

The male-to-female ratio was approximately 3:1, and the mean age ranged between 61.9 and 69.8 years. The mean value of the maximum length of BE ranged between 3 and 8 cm among studies, and in 31.1% of cases, LGD was reported, whereas advanced neoplasia was detected in 64.3% of cases. Two hundred and thirty-five cases of previous BE ablation were reported in eleven studies [16,19,21,24,27,28,29,30,32,34,35], three of which recruited patients with resistant BE following RFA [27,30,32]. Data on the resection of visible lesions were presented in 19 studies with 49.3% of patients warranting a resection of visible lesions prior to ablation. A mean number of 2–5 cryoablation sessions, lasting 6–17 min, were required per study, except for Frederiks et al. [23] who reported a mean number of 7.9 sessions in their multicenter study, whereas in two studies where data for RFA were available, 3–3.5 sessions were required [25,26].

### 3.2. Quality Assessment

Given the absence of randomization, the respective parameters of RoB analysis resulted in a high risk of bias, whereas the attrition and reporting biases were low in the prospective trials. Contrariwise, the retrospective studies preserved a high likelihood of missing data and selection bias, which increased the overall RoB.

Regarding the second arm of this review, all of the cohort studies were graded to have good quality, except for one which was graded as fair [36]. The most frequent issue identified was the lack of a detailed explanation of the statistical methods employed. The study by Alshelleh et al. [36] was rated as fair due to insufficient details about the included cases. However, the results presented in all the studies were sufficient for inclusion in our analysis (Supplementary Table S2).

### 3.3. Primary Outcome

**Figure 3:**
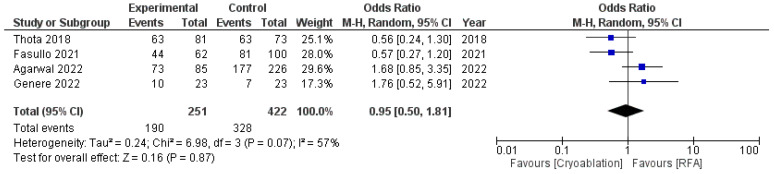
Forest plot reporting odds ratios of CED rates between cryoablation and RFA [14,25,26,27].

Both techniques achieved equal results in terms of CED, with an OR of 0.95 (95%CI: 0.50–1.81) and moderate heterogeneity (I^2^ = 57%, *p* = 0.07) (Figure 3).

**Figure 4:**
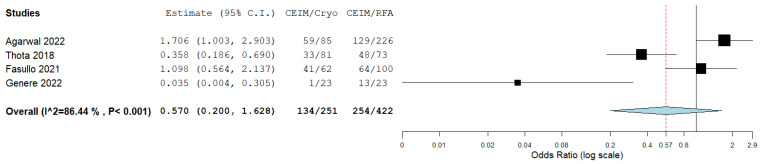
Forest plot reporting odds ratios of CEIM rates between cryoablation and RFA [14,25,26,27].

The complete eradication of intestinal metaplasia did not differ significantly between cryoablation and RFA as well, with an OR of 0.57 (95%CI: 0.20–1.63), albeit with significant heterogeneity (I^2^ = 87%; *p* < 0.001), which was potentially affected by the small sample size in the study by Genere et al. [27] (Figure 4).

### 3.4. Secondary Outcomes

Seventeen studies provided data on CED following the cryoablation of dysplastic BE with a pooled rate of 84.2% (95%CI: 79.1–89.3; I^2^ = 85.8%, *p* < 0.001) (Supplementary Figure S1). Three more studies were included in the analysis for CEIM, resulting in a pooled rate of 64.1% (95%CI: 49.2–79.0), albeit with very high heterogeneity (I^2^ = 98.5%, *p* < 0.001) (Supplementary Figure S2). Recurrence of ΒE was noted in 8.3% of cases after cryoablation (95%CI: 4.7–11.9; I2 = 70.9%, *p* < 0.001); however, the follow-up period was highly variable between the studies (Supplementary Figure S3).

The pooled rates of overall AEs were 14.5% (95%CI: 9.9–19.2; I^2^ = 82.6%, *p* < 0.001) for cryoablation, with the most common one being post-procedure pain (Supplementary Figure S4). Regarding stricture formation, the overall incidence was 6.5% (95%CI: 4.1–9.0; I^2^ = 61.1%, *p* < 0.001); however, only three cases with severe and resistant strictures were reported (Supplementary Figure S5).

### 3.5. Subgroup Analysis

The high heterogeneity, accompanying all outcomes, triggered the subgroup analysis between cryoablation for spray catheters and cryoballoons. Considering CED, the balloon catheter provided higher rates of success [94% (94%CI: 89.4–98.6)] with non-significant (*p* = 0.076), although moderate (I^2^ = 56.5%), heterogeneity compared to the spray catheter, which resulted in lower rates of CED with high heterogeneity [80.2% (95%CI: 73.3–87.1; I^2^ = 86.8%, *p* < 0.001)] (Supplementary Figure S6). The cryoballoon achieved higher rates of CEIM as well [87.2% (95%CI: 80.3–94.2)] with almost half of the cases with spray catheters yielding this outcome [52.7% (95%CI: 29.5–75.8; I^2^ = 98.9%, *p* < 0.001)]; however, heterogeneity remained high in both subgroups (Supplementary Figure S7). Recurrence rates were minimized with the use of balloon catheters [3.9% (95%CI: 0.0–8.6%; I^2^ = 73%, *p* = 0.024)]; however, only spray catheters achieved non-significant heterogeneity [9.9% (95%CI: 6.2–13.7; I^2^ = 41.6%, *p* = 0.081)] (Supplementary Figure S8).

Interestingly, both balloons and spray catheters had similar rates of adverse events [15.8% (95%CI: 11.6–19.9) and 12.1% (95%CI: 5.9–18.3), respectively]; however, only the balloons achieved low heterogeneity (I^2^ = 24.97%, *p* = 0.22) (Supplementary Figure S9). Likewise, in the sub-category of stricture development, both subgroups preserved similar rates to the pooled ones [6% (95%CI: 2.9–9.2) for balloons and 7.5% (95%CI: 3.0–11.9) for the spray catheters], and the cryoballoon subgroup yielded non-significant heterogeneity (I^2^ = 53.7% vs. 70.9%, respectively) (Supplementary Figure S10). Only two studies provided comparative results for this outcome, with Agarwal et al. [14] revealing a significant difference between RFA and cryoablation (4.4% vs. 10.6%, respectively), whereas Genere et al. [27] presented equivalent stricture prevalence.

### 3.6. Sensitivity Analysis

Given the methodological difference between prospective and retrospective design, a sensitivity analysis was conducted to evaluate the impact of the study design on all of our outcomes including only prospective studies. All estimates remained unchanged (Supplementary Figure S11).

### 3.7. Publication Bias

The funnel plot of the primary outcome is symmetrical, thereby suggesting the absence of publication bias (Supplementary Figure S12).

## 4. Discussion

This systematic review with a meta-analysis presents comparative data on the performance of cryoablation and RFA in terms of CED and CEIM and pooled results of the two commercially available cryoablation techniques for patients with BE. The comparison between cryoablation and RFA did not reveal any significant difference in dysplasia or intestinal metaplasia eradication, with an OR of 0.95 and 0.57, respectively. After including the entire literature on cryoablation, the pooled rates of CED and CEID were 84.2% (95%CI: 79.1–89.3) and 64.1% (95%CI: 49.2–79.0), respectively. Adverse events, especially pain, were recorded at a rate of 14.5% (95%CI: 9.9–19.2), and, wherever follow-up was provided, strictures were diagnosed in 6.5% (95%CI: 4.1–9.0) and recurrences in 8.3% (95%CI: 4.7–11.9) of cases. The main strength of this meta-analysis is that it compares the performance of cryoablation with the “gold-standard” RFA and, also, provides cumulative evidence based on the entire spectrum of available data. However, all of the outcomes were accompanied by high heterogeneity scores, probably due to the design and the power of the included studies.

Cryoablation was, at least, non-inferior to RFA in our analysis in terms of efficacy, which confirms the results from Gomes et al. [37], who included a subset of studies and reported no difference in CEIM (risk difference: −0.03; 95%CI: −0.25–0.19) and CED (risk difference: −0.03; 95%CI: −0.15–0.09). The absolute number of adverse events was low, and there was no difference in the recurrence rate [37]. The resulting pooled CED rates in our meta-analysis were 84.2% (95%CI: 79.1–89.3), and CEIM was achieved in 64.1% (95%CI: 49.2–79.0) of cases. Likewise, a meta-analysis of nineteen studies evaluating RFA revealed rates of 91% (95%CI: 87–95) for CED and 78% (95%CI: 70–86) for CEIM [38]. After eradication with RFA, BE recurred in 13% (95%CI: 9–18) compared with 8.3% (95%CI: 4.7–11.9) of cases following cryoablation, although no direct comparison can be made. A network meta-analysis of 23 RCTs, which compared RFA, APC, PDT, and surveillance in BE, indicated that RFA achieved surfaces under cumulative ranking (SUCRA) values of 60.8% and 74.4% for CED and CEIM, respectively, with APC achieving the highest eradication rates of metaplastic mucosa and PDT the highest CED (94.1%) [10]. A respective meta-analysis calculated the pooled rates of APC performance and safety, revealing that CEIM was achieved in 86.8% (95%CI: 83.5–90.2) of cases, with high power and hAPC having a higher rate compared to standard APC. The pooled recurrence rate was 16.1% (95%CI: 10.7–21.6), which is higher than our results [8.3% (95%CI: 4.7–11.9)], although more studies providing a direct comparison are required [39]. Although a competent therapeutic endoscopist can perform both RFA and cryoablation without significant difficulty, there are some differences between these techniques that should be considered. First, RFA devices are not controlled through the scope, albeit the focal ones are mounted over the scope, whereas both the cryoballoon and the catheter are controlled through the scope. In cases of circumferential BE, a 360 RFA ablation catheter might be more feasible to apply compared to the focal ablation (areas measuring 2 to 3 cm^2^ are treated until the entire BE is reached) provided by cryoablation devices. A significant parameter in the era of green endoscopy is the consumed energy; however, comparative data are absent.

One of the main considerations of ablative therapies is the rate of adverse events, especially stricture formation. Cryoablation was associated with AEs in 14.5% (95%CI: 9.9–19.2) of patients, and the studies using balloons provided similar and homogenous results, whereas the use of spray catheters was associated with more variable results. Likewise, both subtypes had similar rates of stricture formation with pooled rates of 6.5% (95%CI: 4.1–9.0); however, the cryoballoon yielded moderate and insignificant heterogeneity (I^2^ = 53.7%), and only three cases with severe strictures requiring multiple dilatations were identified. Stricture formation pooled rates were similar for RFA in a respective meta-analysis [38] [5% (95%CI: 3–7)], and according to Gomes et al. [37], these techniques did not differ significantly [risk difference = 0.09 (95%CI: −0.02–0.19)]. On the other hand, APC was associated with higher rates of adverse events [22.5% (95%CI: 15.3–29.7)], albeit with minimum stricture development [1.7% (95%CI: 0.9–2.6)] [39]. These data indicate that cryoablation, especially the use of cryoballoons, represents a procedure that is equally as safe as the other alternatives.

The recorded heterogeneity was significant for all outcomes in the pooled analysis, and therefore a subgroup analysis was performed to investigate if the type of ablation device was responsible for heterogeneity. Studies assessing cryoballoons achieved a reduction in heterogeneity for all outcomes, except for CEIM and BE recurrence. More specifically, CED was more common after treatment with a cryoballoon with lower and non-significant heterogeneity compared to the pooled results and the spray catheter. Regarding CEIM, the heterogeneity was equally high in both subgroups, but the cryoballoon was associated with higher rates of success compared to spray catheters, with only 52.7% (95%CI: 29.5–75.8) of cases yielding eradication. A potential explanation for this discrepancy is the larger surface that, theoretically, the balloon could cover, although studies associating the ablated surface with the final outcome do not exist. This outcome was homogenously preserved in about 90% of cases using spray catheters, whereas heterogeneity remained high (I^2^ = 73%) when using the balloon catheter, despite the low recurrence rate [3.9% (95%CI: 0.0–8.6%)].

This study, although it accumulates the entire literature on cryoablation, had some limitations. First, there are no RCTs evaluating this approach, thereby reducing the quality of evidence. The available types of studies were prospective and retrospective ones, and to investigate the potential impact of the design on our results, we performed a sensitivity analysis, excluding retrospective studies, which did not reveal changes in the results. Moreover, only four studies provided comparisons between cryoablation and RFA, thus limiting the strength of the direct comparison of these approaches. Further studies, especially RCTs, demonstrating this comparison, and a direct comparison between cryoablation, APC, and PDT, are expected to provide more evidence to allow for a clear view of these approaches. Lastly, potential variables that could influence CEM, CEIM, stricture development, or recurrence (such as the indication for transplantation) were not evaluated in the included studies and, therefore, were not considered in this meta-analysis.

## 5. Conclusions

To conclude, cryoablation represents an efficient and safe approach in BE management, according to a direct comparison with RFA and the cumulative performance rates. Current evidence cannot prove superiority over the other alternatives; however, the use of cryoballoons seems to provide less variable results compared to spray catheter use. Future comparisons with the entire spectrum of ablative techniques, including cost-effectiveness analyses, are necessary to guide the optimal approach selection in clinical practice.

## Figures and Tables

**Table 1:** Main characteristics of included studies.

						Patients									
	First Author	Year	Country	Study Design	Recruitment Period	Cryoablation	RFA	Type of Device	Male	Age	Mean Maximum Length	LGD	HGD/OC	Sessions	Prior EMR
1	Snady	2023	USA	Prospective	n/a	62	-	Spray catheter	32	62.2	3	16	11	2.8	
2	Agarwal	2022	USA	Retrospective	2014–2020	85	226	Cryoballoon	148	67.1	3.6	32	53	-	51
3	Overwater	2021	Netherlands	Prospective	2018–2020	25	-	Cryoballoon	23	69	3	10	15	-	14
4	Canto	2020	USA	Multicenter prospective	2016–2018	94	-	Cryoballoon	76	65	3.1	19	73	2	40
5	van Munster	2019	Netherlands	Prospective	2017–2018	25	-	Cryoballoon	19	67	3	19	5	-	5
6	van Munster	2018	Netherlands	Prospective	2016–2017	20	26	Cryoballoon	38	66	2	9	11	-	10
7	Canto	2018	USA	Prospective	2015–2016	41	-	Cryoballoon	34	65.7	3.9	13	28	3	14
8	Künzli	2016	Netherlands	Prospective	2015–2016	30	-	Cryoballoon	26	66	2	14	16	-	15
9	Schölvinck	2015	Netherlands	Multicenter prospective	2012–2014	39	-	Cryoballoon	35	66	5	9	20	-	12
10	Frederiks	2022	Multicenter	Retrospective	2019–2020	56	-	Cryoballoon	49	67–68	2	16	40	7.9	
11	Eluri	2024	USA	Multicenter prospective	2013–2022	138	-	Spray catheter	106	69	3.6	33	105	-	62
12	Thota	2018	USA	Prospective	2006–2011	81	73	Spray catheter	131	69.8	5.2	13	60	3	35
13	Fasullo	2021	USA	Retrospective	2014–2020	62	100	Spray catheter	143	67.1	4.7	36	26	4.8	
14	Genere	2022	USA	Retrospective	2005–2020	23	23	Spray catheter	33	68	8	7	16	4	13
15	Dumot	2009	USA	Prospective	2005–2008	30	-	Spray catheter	21	69.7	6.1	-	30	5	1
16	Shaheen	2011	USA	Multicenter retrospective		60	-	Spray catheter	50	64	5	-	-	4	15
17	Sengupta	2015	USA	Retrospective	2006–2013	16	-	Spray catheter	-	-	-	6	9	3	3
18	Ramay	2017	USA	Prospective	2006–2012	50	-	Spray catheter	47	61.9	3.5		-	3	14
19	Trindade	2017	USA	Multicenter prospective	2008–2014	18	-	Spray catheter	15	64.5	4	7	11	3	3
20	Trindade	2018	USA	Multicenter retrospective	2007–2015	27	-	Spray catheter	24	68	5	0	27	3	27
21	Spiceland	2019	USA	Retrospective	2007–2018	46	-	Both spray catheter and cryoballoon	42	66	>3	15	31	2	23
22	Kaul	2020	USA	Prospective	2008–2019	57	-	Spray catheter	51	-	6.2	8	49	3	39
23	Alshelleh	2021	USA	Retrospective	2015–2019	71	-	Both spray catheter and cryoballoon	59	65.6	3.2	34	37	-	36

## Supplementary Materials

The following supporting information can be downloaded at: https://www.mdpi.com/article/10.3390/cancers16172937/s1, Table S1: PRISMA 2020 checklist of the presented objects in this review; Table S2: Risk of bias assessment; Figure S1. Pooled rates of CED after cryoablation; Figure S2. Pooled rates of CEIM after cryoablation; Figure S3. Pooled rates of recurrence of BO after successful cryoablation; Figure S4. Pooled rates of overall adverse events after cryoablation; Figure S5. Pooled rates of stricture formation after cryoablation; Figure S6. Subgroup analysis of CED after cryoablation with (a) spray catheter (b) cryoballoon; Figure S7. Subgroup analysis of CEIM after cryoablation with (a) spray catheter (b) cryoballoon; Figure S8. Subgroup analysis of recurrence of BO after successful cryoablation with (a) spray catheter (b) cryoballoon; Figure S9. Subgroup analysis of overall adverse events after cryoablation with (a) spray catheter (b) cryoballoon; Figure S10. Subgroup analysis of stricture formation after cryoablation with (a) spray catheter (b) cryoballoon; Figure S11. Sensitivity analysis of CED after excluding retrospective studies; Figure S12. Funnel plot illustrating the absence of publication bias of the analysis concerning the primary outcome.
