# Supplementary material for: Efficacy and Safety of Cryoablation in Barrett’s Esophagus and Comparison with Radiofrequency Ablation: A Meta-Analysis
Source: Cancers (Basel). 2024 Aug 23;16(17):2937. doi: 10.3390/cancers16172937 (PMC11394299; doi:10.3390/cancers16172937)
Supplement: Supplementary file 1 [file cancers-16-02937-s001.zip › cancers-3143246-supplementary.pdf]

**Supplementary Materials**

**Supplementary Table S1** PRISMA 2020 checklist of the presented objects in this review

| Section and Topic | Item # | Checklist item | Reported (Yes/No) |
| --- | --- | --- | --- |
| **TITLE** | | | |
| Title | 1 | Identify the report as a systematic review. | YES/p1 |
| **ABSTRACT BACKGROUND** | | | |
| Objectives | 2 | Provide an explicit statement of the main objective(s) or question(s) the review addresses. | YES/p3 |
| **ABSTRACT METHODS** | | | |
| Eligibility criteria | 3 | Specify the inclusion and exclusion criteria for the review. | YES/p3 |
| Information sources | 4 | Specify the information sources (e.g., databases, registers) used to identify studies and the date when OACh was last searched. | YES/p3 |
| Risk of bias | 5 | Specify the methods used to assess risk of bias in the included studies. | YES/p3 |
| Synthesis of results | 6 | Specify the methods used to present and synthesise results. | YES/p3 |
| **ABSTRACT RESULTS** | | | |
| Included studies | 7 | Give the total number of included studies and participants and summarise relevant characteristics of studies. | YES/p3 |
| Synthesis of results | 8 | Present results for main outcomes, preferably indicating the number of included studies and participants for OACh. If meta-analysis was done, report the summary estimate and confidence/credible interval. If comparing groups, indicate the direction of the effect (i.e. which group is favoured). | YES/p3 |
| **ABSTRACT DISCUSSION** | | | |
| Limitations of evidence | 9 | Provide a brief summary of the limitations of the evidence included in the review (e.g., study risk of bias, inconsistency and imprecision). | YES/p3 |
| Interpretation | 10 | Provide a general interpretation of the results and important implications. | YES/p3 |

Study checklist

| Section and Topic | Item # | Checklist item | Location where item is reported |
|---|---|---|---|
| **TITLE** | | | |
| Title | 1 | Identify the report as a systematic review. | Page 1 |
| **ABSTRACT** | | | |
| Abstract | 2 | See the PRISMA 2020 for Abstracts checklist. | Page 3 |
| **INTRODUCTION** | | | |
| Rationale | 3 | Describe the rationale for the review in the context of existing knowledge. | Pages 4 |
| Objectives | 4 | Provide an explicit statement of the objective(s) or question(s) the review addresses. | Page 5 |
| **METHODS** | | | |
| Eligibility criteria | 5 | Specify the inclusion and exclusion criteria for the review and how studies were grouped for the syntheses. | Page 6 |
| Information sources | 6 | Specify all databases, registers, websites, organizations, reference lists and other sources searched or consulted to identify studies. Specify the date when OACh source was last searched or consulted. | Pages 6-7 |
| Search strategy | 7 | Present the full search strategies for all databases, registers and websites, including any filters and limits used. | Pages 6-7 |
| Selection process | 8 | Specify the methods used to decide whether a study met the inclusion criteria of the review, including how many reviewers screened OACh record and OACh report retrieved, whether they worked independently, and if applicable, details of automation tools used in the process. | Page 7 |
| Data collection process | 9 | Specify the methods used to collect data from reports, including how many reviewers collected data from OACh report, whether they worked independently, any processes for obtaining or confirming data from study investigators, and if applicable, details of automation tools used in the process. | Page 7 |

| Section and Topic | Item # | Checklist item | Location where item is reported |
|---|---|---|---|
| Data items | 10a | List and define all outcomes for which data were sought. Specify whether all results that were compatible with OACh outcome domain in OACh study were sought (e.g., for all measures, time points, analyses), and if not, the methods used to decide which results to collect. | Page 7-8 |
| | 10b | List and define all other variables for which data were sought (e.g., participant and intervention characteristics, funding sources). Describe any assumptions made about any missing or unclear information. | Pages 7-8 |
| Study risk of bias assessment | 11 | Specify the methods used to assess risk of bias in the included studies, including details of the tool(s) used, how many reviewers assessed OACh study and whether they worked independently, and if applicable, details of automation tools used in the process. | Page 7 |
| Effect measures | 12 | Specify for OACh outcome the effect measure(s) (e.g., risk ratio, mean difference) used in the synthesis or presentation of results. | Page 7 |
| Synthesis methods | 13a | Describe the processes used to decide which studies were eligible for OACh synthesis (e.g., tabulating the study intervention characteristics and comparing against the planned groups for OACh synthesis (item #5)). | Pages 8 |
| | 13b | Describe any methods required to prepare the data for presentation or synthesis, such as handling of missing summary statistics, or data conversions. | Pages 8 |
| | 13c | Describe any methods used to tabulate or visually display results of individual studies and syntheses. | Pages 8 |
| | 13d | Describe any methods used to synthesize results and provide a rationale for the choice(s). If meta-analysis was performed, describe the model(s), method(s) to identify the presence and extent of statistical heterogeneity, and software package(s) used. | Pages 8 |
| | 13e | Describe any methods used to explore possible causes of heterogeneity among study results (e.g., subgroup analysis, meta-regression). | Page 8 |
| | 13f | Describe any sensitivity analyses conducted to assess robustness of the synthesized results. | Page 8 |
| Reporting bias assessment | 14 | Describe any methods used to assess risk of bias due to missing results in a synthesis (arising from reporting biases). | Page 7 |

| Section and Topic | Item # | Checklist item | Location where item is reported |
|---|---|---|---|
| Certainty assessment | 15 | Describe any methods used to assess certainty (or confidence) in the body of evidence for an outcome. | |
| **RESULTS** | | | |
| Study selection | 16a | Describe the results of the search and selection process, from the number of records identified in the search to the number of studies included in the review, ideally using a flow diagram. | Page 9, Figure 1 |
| | 16b | Cite studies that might appear to meet the inclusion criteria, but which were excluded, and explain why they were excluded. | Page 9, Figure 1 |
| Study characteristics | 17 | Cite OACh included study and present its characteristics. | Pages 9-10, Table 1 |
| Risk of bias in studies | 18 | Present assessments of risk of bias for OACh included study. | Pages 10-11 and suppl Table 2 |
| Results of individual studies | 19 | For all outcomes, present, for OACh study: (a) summary statistics for OACh group (where appropriate) and (b) an effect estimate and its precision (e.g., confidence/credible interval), ideally using structured tables or plots. | Page 11 |
| Results of syntheses | 20a | For OACh synthesis, briefly summarise the characteristics and risk of bias among contributing studies. | Page 10, suppl table 2 |
| | 20b | Present results of all statistical syntheses conducted. If meta-analysis was done, present for OACh the summary estimate and its precision (e.g., confidence/credible interval) and measures of statistical heterogeneity. If comparing groups, describe the direction of the effect. | Pages 11-12, figure 2 and suppl fig 1-11 |
| | 20c | Present results of all investigations of possible causes of heterogeneity among study results. | Page 12, suppl fig 5-9 |
| | 20d | Present results of all sensitivity analyses conducted to assess the robustness of the synthesized results. | Page 13, suppl fig 9 |

| Section and Topic | Item # | Checklist item | Location where item is reported |
|---|---|---|---|
| Reporting biases | 21 | Present assessments of risk of bias due to missing results (arising from reporting biases) for OACh synthesis assessed. | Suppl fig 12 |
| Certainty of evidence | 22 | Present assessments of certainty (or confidence) in the body of evidence for OACh outcome assessed. | |
| **DISCUSSION** | | | |
| Discussion | 23a | Provide a general interpretation of the results in the context of other evidence. | Page 13-16 |
| | 23b | Discuss any limitations of the evidence included in the review. | Page 15 |
| | 23c | Discuss any limitations of the review processes used. | Page 15 |
| | 23d | Discuss implications of the results for practice, policy, and future research. | Page 15-16 |

**Supplementary Table S2** Risk of bias assessment

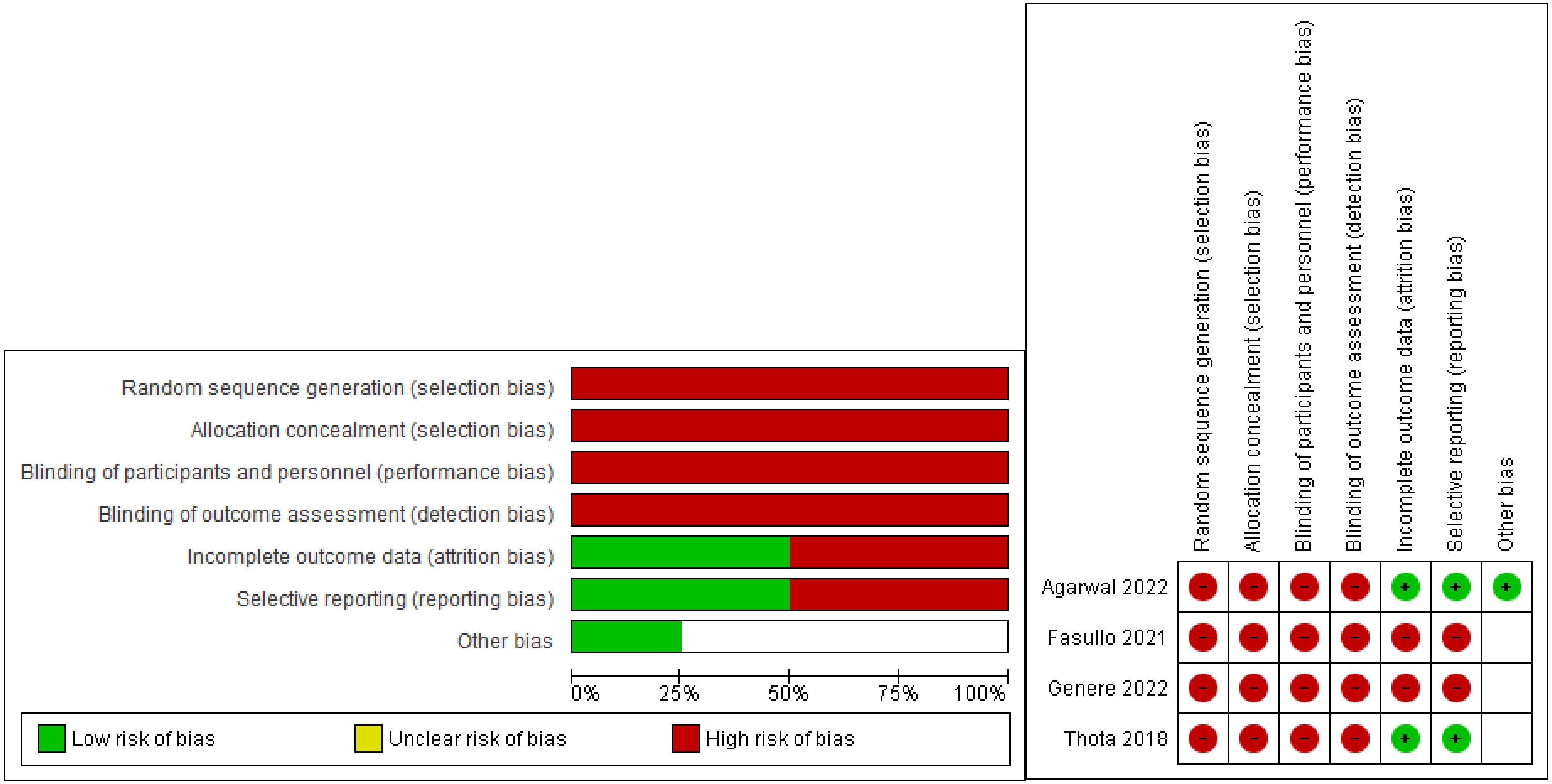

**The results of quality assessment based on the National Heart, Lung, and Blood Institute tool for case-series**

| Studies | Quality assessment | | | | | | | | |
|---|---|---|---|---|---|---|---|---|---|
| **Author, year** | **Was the study question or objective clearly stated?** | **Was the study population clearly and fully described, including a case definition?** | **Were the cases consecutive?** | **Were the subjects comparable?** | **Was the intervention clearly described?** | **Were the outcome measures clearly defined, valid, reliable, and implemented consistently across all study participants?** | **Was the length of follow-up adequate?** | **Were the statistical methods well-described?** | **Were the results well-described?** | **Quality rating (Good, Fair, Poor)** |
| Dumot et al | YES | YES | YES | YES | YES | YES | YES | YES | YES | GOOD |
| Shaheen et al | YES | YES | YES | YES | YES | YES | YES | YES | YES | GOOD |
| Schölvinck et al | YES | YES | YES | YES | YES | YES | YES | YES | YES | GOOD |
| Sengupta et al | YES | YES | NO | YES | YES | YES | YES | YES | YES | GOOD |
| Künzli et al | YES | YES | YES | YES | YES | YES | YES | NO | YES | GOOD |
| Ramay et al | YES | YES | YES | YES | YES | YES | YES | NO | YES | GOOD |
| Trindade (1) et al | YES | YES | YES | YES | YES | YES | YES | NO | YES | GOOD |
| Canto (2) et al | YES | YES | YES | YES | YES | YES | YES | YES | YES | GOOD |
| Trindade (2) et al | YES | YES | YES | YES | YES | YES | YES | NO | YES | GOOD |
| Van Munster (1) et al | YES | YES | YES | YES | YES | YES | YES | YES | YES | GOOD |
| Spiceland et al | YES | YES | YES | YES | YES | YES | YES | NO | YES | GOOD |
| Canto (1) et al | YES | YES | YES | YES | YES | YES | YES | YES | YES | GOOD |
| Kaul et al | YES | YES | YES | YES | YES | YES | YES | NO | YES | GOOD |
| Alshelleh et al | YES | NO | NO | YES | NO | YES | YES | NO | YES | FAIR |
| Overwater et al | YES | YES | YES | YES | YES | YES | YES | NO | YES | GOOD |
| Frederiks et al | YES | YES | YES | YES | YES | YES | YES | YES | YES | GOOD |
| Snady et al | YES | YES | YES | YES | YES | YES | YES | YES | YES | GOOD |
| Eluri et al | YES | YES | YES | YES | YES | YES | YES | YES | YES | GOOD |

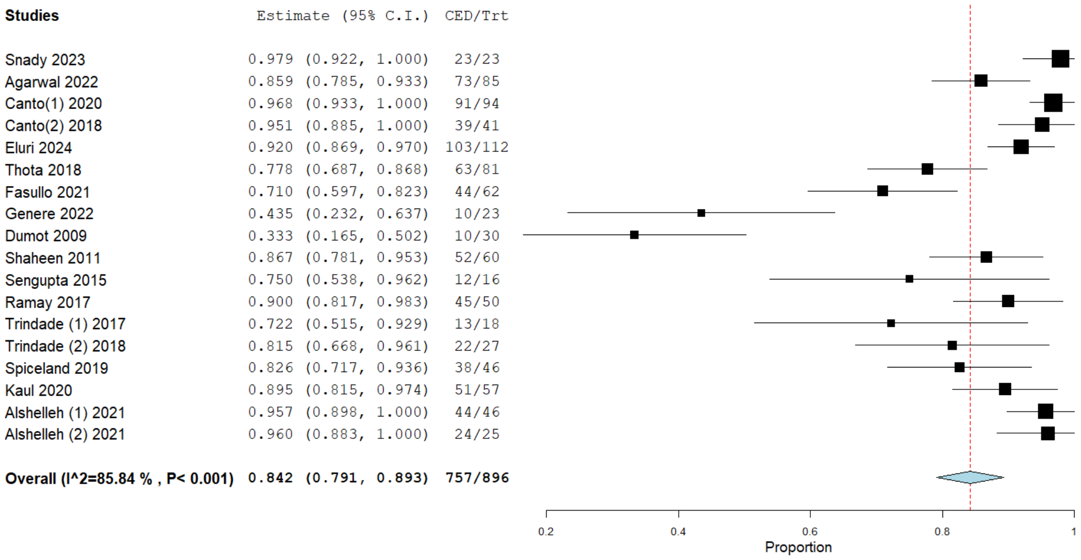


**Supplementary Figure S1**. Pooled rates of CED after cryoablation

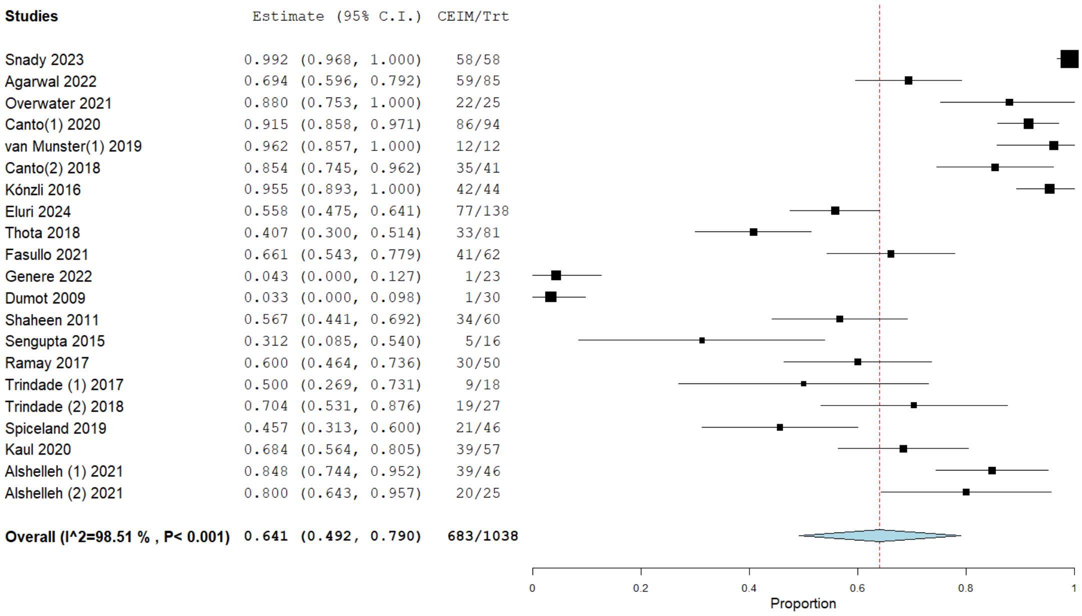


**Supplementary Figure S2**. Pooled rates of CEIM after cryoablation

| Studies | Estimate (95% C.I.) | Recurrencies/Trt |
|---|---|---|
| Snady 2023 | 0.081 (0.013, 0.148) | 5/62 |
| Agarwal 2022 | 0.106 (0.040, 0.171) | 9/85 |
| Canto(1) 2020 | 0.011 (0.000, 0.031) | 1/94 |
| Canto(2) 2018 | 0.024 (0.000, 0.072) | 1/41 |
| Eluri 2024 | 0.088 (0.021, 0.156) | 6/68 |
| Thota 2018 | 0.143 (0.056, 0.229) | 9/63 |
| Fasullo 2021 | 0.136 (0.035, 0.238) | 6/44 |
| Genere 2022 | 0.348 (0.153, 0.542) | 8/23 |
| Sengupta 2015 | 0.029 (0.000, 0.110) | 0/16 |
| Ramay 2017 | 0.133 (0.012, 0.255) | 4/30 |
| Trindade (1) 2017 | 0.026 (0.000, 0.098) | 0/18 |
| Trindade (2) 2018 | 0.111 (0.000, 0.230) | 3/27 |
| Kaul 2020 | 0.123 (0.038, 0.208) | 7/57 |
| **Overall (I^2=70.93 % , P< 0.001)** | **0.083 (0.047, 0.119)** | **59/628** |

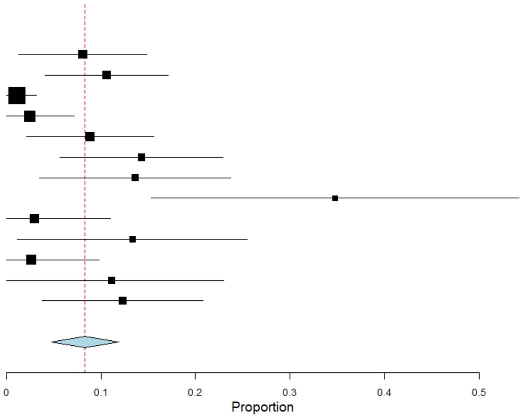


**Supplementary Figure S3.** Pooled rates of recurrence of BO after successful cryoablation.

| Studies | Estimate (95% C.I.) | Adverse Events/Trt |
|---|---|---|
| Snady 2023 | 0.339 (0.221, 0.457) | 21/62 |
| Agarwal 2022 | 0.106 (0.040, 0.171) | 9/85 |
| Overwater 2021 | 0.240 (0.073, 0.407) | 6/25 |
| Canto(1) 2020 | 0.170 (0.094, 0.246) | 16/94 |
| van Munster(1) 2019 | 0.200 (0.043, 0.357) | 5/25 |
| Canto(2) 2018 | 0.244 (0.112, 0.375) | 10/41 |
| Kónzli 2016 | 0.067 (0.000, 0.156) | 2/30 |
| Eluri 2024 | 0.022 (0.000, 0.046) | 3/138 |
| Genere 2022 | 0.174 (0.019, 0.329) | 4/23 |
| Dumot 2009 | 0.400 (0.225, 0.575) | 12/30 |
| Shaheen 2011 | 0.100 (0.024, 0.176) | 6/60 |
| Sengupta 2015 | 0.375 (0.138, 0.612) | 6/16 |
| Trindade (1) 2017 | 0.026 (0.000, 0.098) | 0/18 |
| Trindade (2) 2018 | 0.018 (0.000, 0.067) | 0/27 |
| Kaul 2020 | 0.035 (0.000, 0.083) | 2/57 |
| van Munster (2) 2018 | 0.250 (0.060, 0.440) | 5/20 |
| Schφlvinck 2015 | 0.154 (0.041, 0.267) | 6/39 |
| Frederiks 2022 | 0.196 (0.092, 0.300) | 11/56 |
| **Overall (I^2=82.56 % , P< 0.001)** | **0.145 (0.099, 0.192)** | **124/846** |

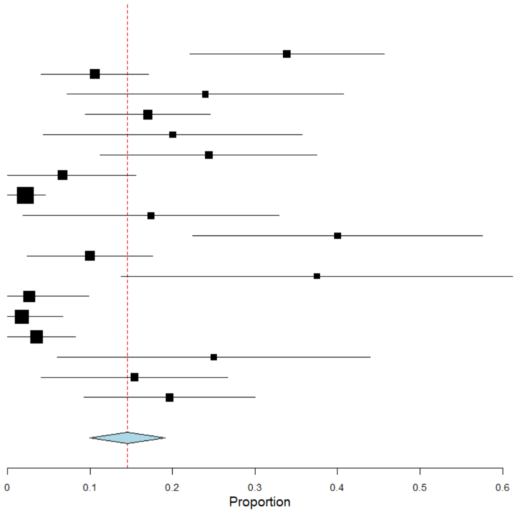


**Supplementary Figure S4**. Pooled rates of overall adverse events after cryoablation

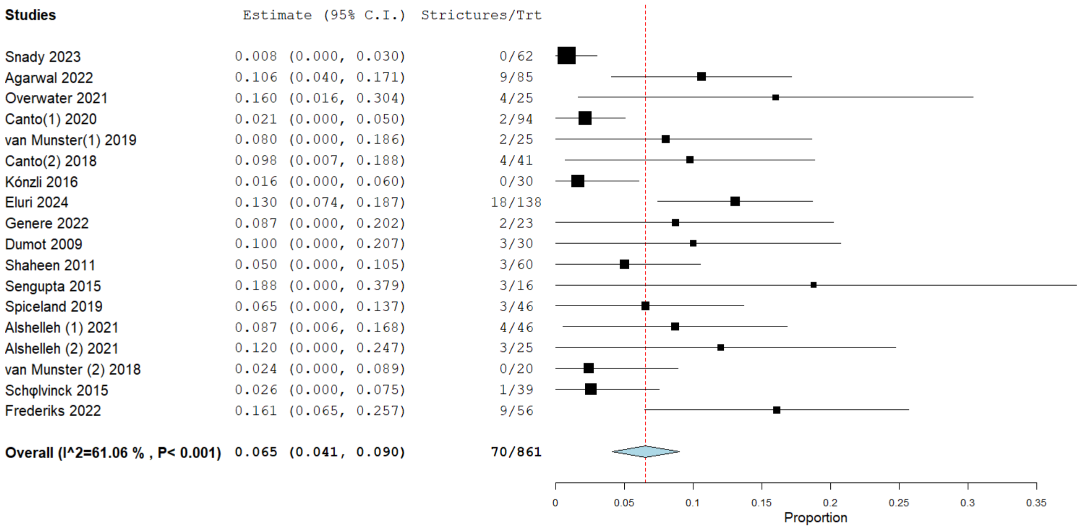


**Supplementary Figure S5.** Pooled rates of stricture formation after cryoablation

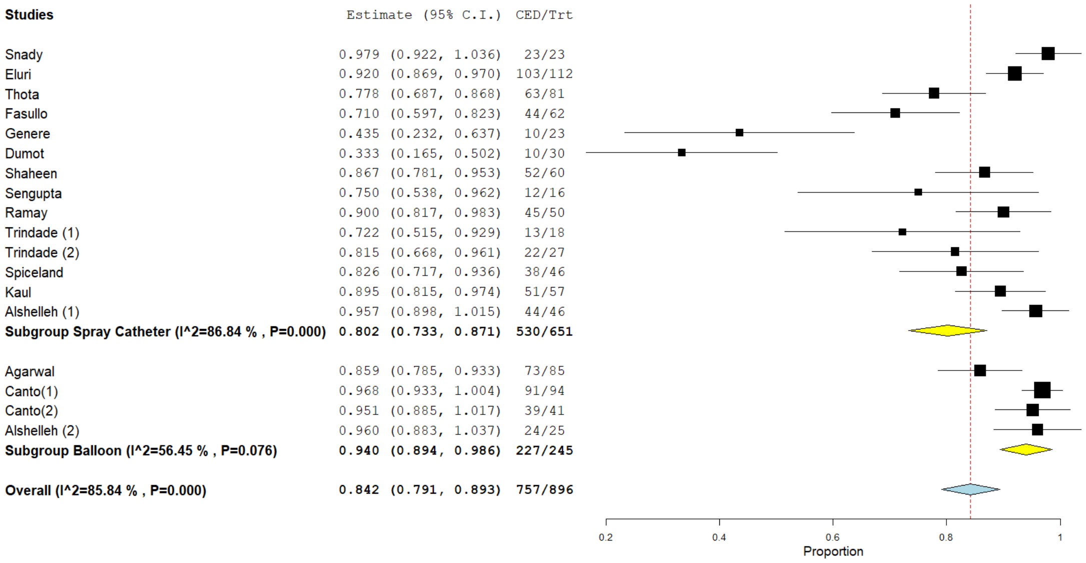


**Supplementary Figure S6.** Subgroup analysis of CED after cryoablation with (a) spray catheter (b) cryoballoon

| Studies | Estimate (95% C.I.) | CEIM/Trt |
|---|---|---|
| Snady | 0.992 (0.968, 1.015) | 58/58 |
| Eluri | 0.558 (0.475, 0.641) | 77/138 |
| Thota | 0.407 (0.300, 0.514) | 33/81 |
| Fasullo | 0.661 (0.543, 0.779) | 41/62 |
| Genere | 0.043 (−0.040, 0.127) | 1/23 |
| Dumot | 0.033 (−0.031, 0.098) | 1/30 |
| Shaheen | 0.567 (0.441, 0.692) | 34/60 |
| Sengupta | 0.312 (0.085, 0.540) | 5/16 |
| Ramay | 0.600 (0.464, 0.736) | 30/50 |
| Trindade (1) | 0.500 (0.269, 0.731) | 9/18 |
| Trindade (2) | 0.704 (0.531, 0.876) | 19/27 |
| Spiceland | 0.457 (0.313, 0.600) | 21/46 |
| Kaul | 0.684 (0.564, 0.805) | 39/57 |
| Alshelleh (1) | 0.848 (0.744, 0.952) | 39/46 |
| **Subgroup Spray Catheter (I^2=98.98 % , P=0.000)** | **0.527 (0.295, 0.758)** | **407/712** |
| Agarwal | 0.694 (0.596, 0.792) | 59/85 |
| Overwater | 0.880 (0.753, 1.007) | 22/25 |
| Canto(1) | 0.915 (0.858, 0.971) | 86/94 |
| van Munster(1) | 0.962 (0.857, 1.066) | 12/12 |
| Canto(2) | 0.854 (0.745, 0.962) | 35/41 |
| Kónzli | 0.955 (0.893, 1.016) | 42/44 |
| Alshelleh (2) | 0.800 (0.643, 0.957) | 20/25 |
| **Subgroup Balloon (I^2=74.79 % , P=0.001)** | **0.872 (0.803, 0.942)** | **276/326** |
| **Overall (I^2=98.51 % , P=0.000)** | **0.641 (0.492, 0.790)** | **683/1038** |

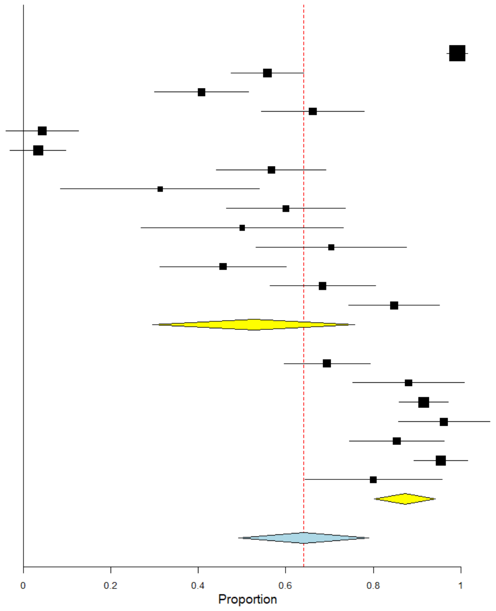


**Supplementary Figure S7.** Subgroup analysis of CEIM after cryoablation with (a) spray catheter (b) cryoballoon

| Studies | Estimate (95% C.I.) | Recurrencies/Trt |
|---|---|---|
| Snady | 0.081 (0.013, 0.148) | 5/62 |
| Eluri | 0.088 (0.021, 0.156) | 6/68 |
| Thota | 0.143 (0.056, 0.229) | 9/63 |
| Fasullo | 0.136 (0.035, 0.238) | 6/44 |
| Genere | 0.348 (0.153, 0.542) | 8/23 |
| Sengupta | 0.029 (−0.051, 0.110) | 0/16 |
| Ramay | 0.133 (0.012, 0.255) | 4/30 |
| Trindade (1) | 0.026 (−0.046, 0.098) | 0/18 |
| Trindade (2) | 0.111 (−0.007, 0.230) | 3/27 |
| Kaul | 0.123 (0.038, 0.208) | 7/57 |
| **Subgroup Spray Catheter (I^2=41.55 % , P=0.081)** | **0.099 (0.062, 0.137)** | **48/408** |
| Agarwal | 0.106 (0.040, 0.171) | 9/85 |
| Canto(1) | 0.011 (−0.010, 0.031) | 1/94 |
| Canto(2) | 0.024 (−0.023, 0.072) | 1/41 |
| **Subgroup Balloon (I^2=73.11 % , P=0.024)** | **0.039 (−0.009, 0.086)** | **11/220** |
| **Overall (I^2=70.93 % , P=0.000)** | **0.083 (0.047, 0.119)** | **59/628** |

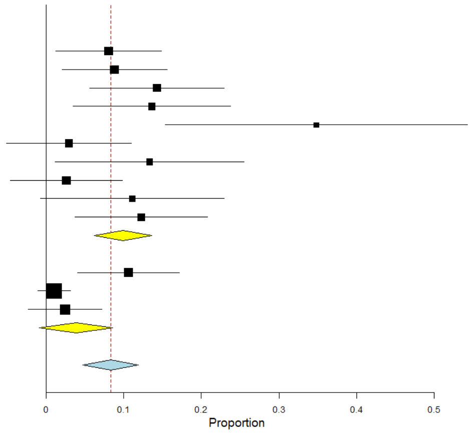


**Supplementary Figure S8.** Subgroup analysis of recurrence of BO after successful cryoablation with (a) spray catheter (b) cryoballoon

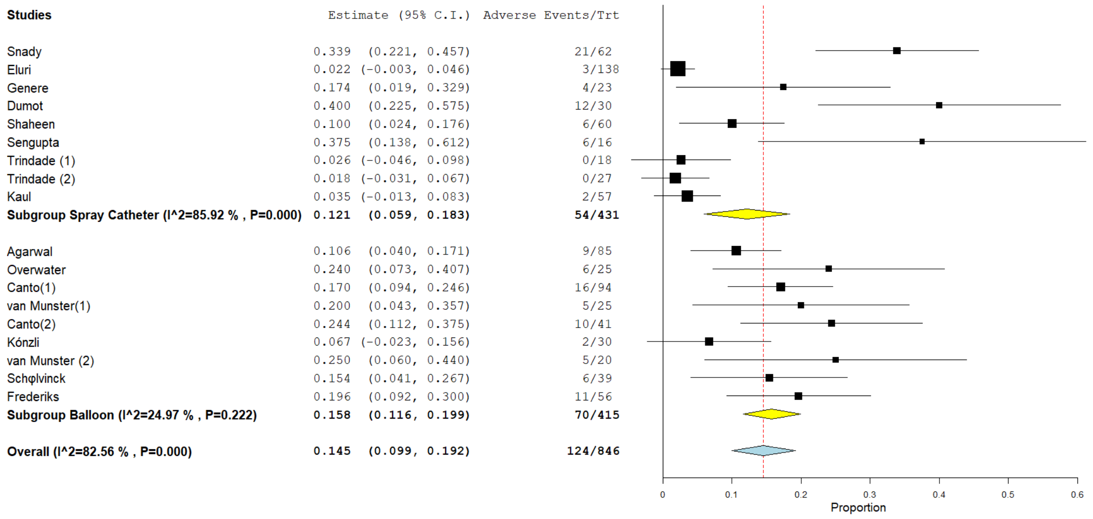


**Supplementary Figure S9.** Subgroup analysis of overall adverse events after cryoablation with (a) spray catheter (b) cryoballoon

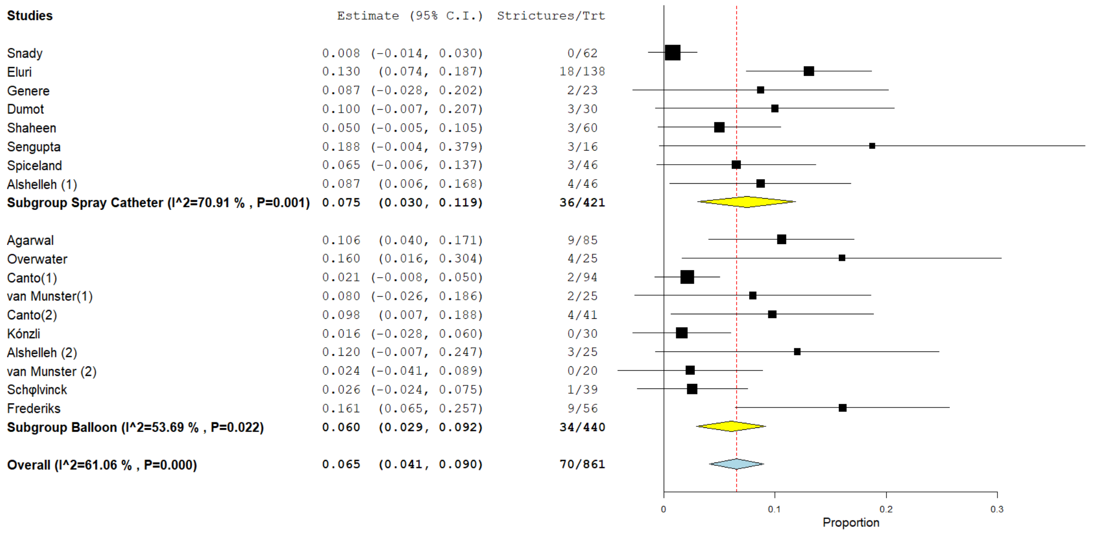


**Supplementary Figure S10.** Subgroup analysis of stricture formation after cryoablation with (a) spray catheter (b) cryoballoon

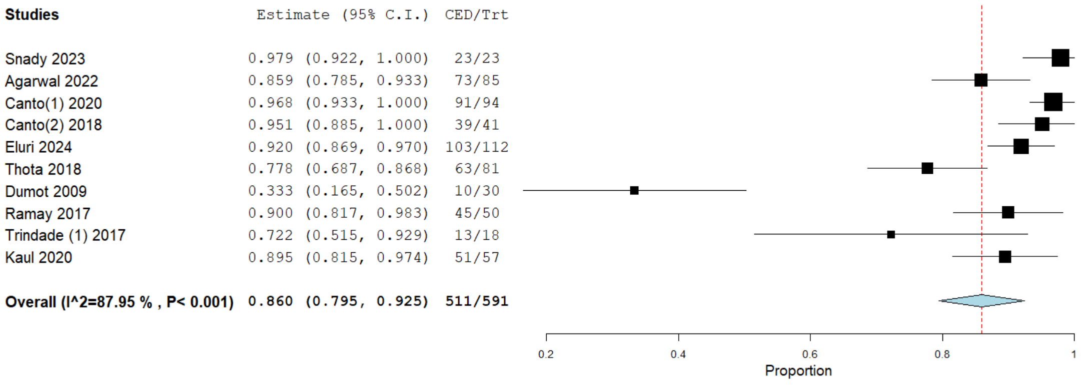


**Supplementary Figure S11.** Sensitivity analysis of CED after excluding retrospective studies.

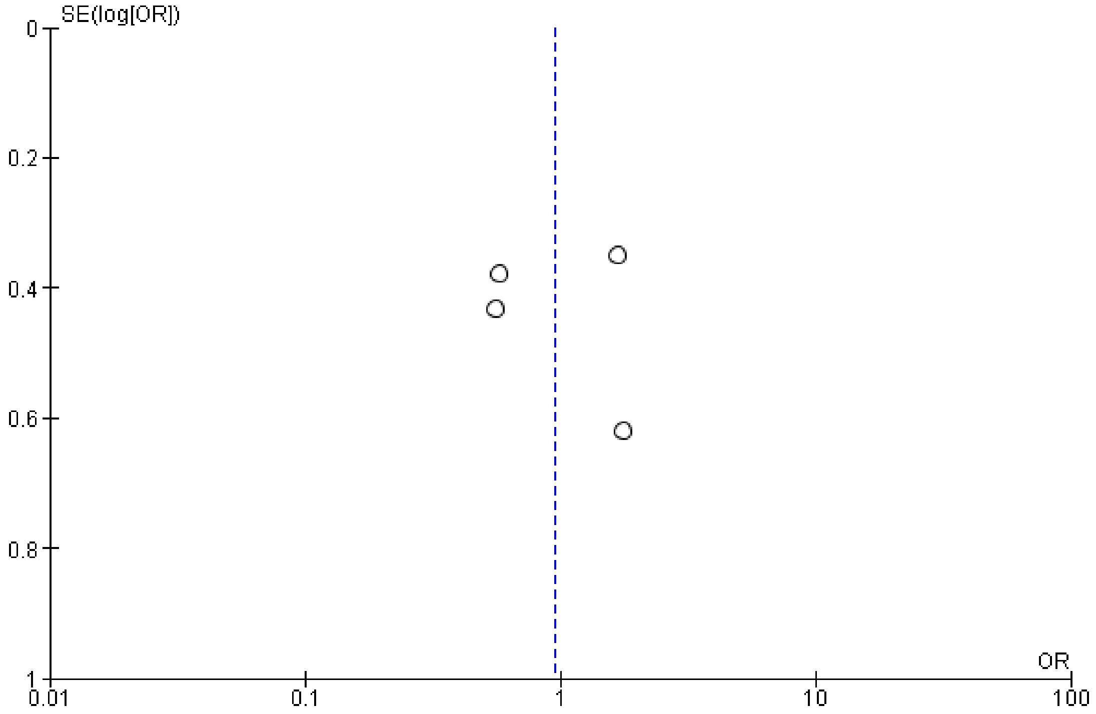


**Supplementary Figure S12.** Funnel plot illustrating the absence of publication bias of the analysis concerning the primary outcome
